# Extracellular and Intracellular Factors in Brain Cancer

**DOI:** 10.3389/fcell.2021.699103

**Published:** 2021-08-27

**Authors:** Kouminin Kanwore, Piniel Alphayo Kambey, Xiao-Xiao Guo, Ayanlaja Abdulrahman Abiola, Ying Xia, Dianshuai Gao

**Affiliations:** Department of Neurobiology and Anatomy, Xuzhou Key Laboratory of Neurobiology, Xuzhou Medical University, Xuzhou, China

**Keywords:** cytoskeleton, endoplasmic reticulum, mitochondria, metabolism, SOX1, doublecortin, autophagy

## Abstract

The external and internal factors of the cell are critical to glioma initiation. Several factors and molecules have been reported to be implicated in the initiation and progression of brain cancer. However, the exact sequence of events responsible for glioma initiation is still unknown. Existing reports indicate that glioma stem cells are the cell of glioma origin. During cell division, chromosome breakage, DNA alteration increases the chance of cell genome modifications and oncogene overexpression. Although there is a high risk of gene alteration and oncogene overexpression, not everyone develops cancer. During embryogenesis, the same oncogenes that promote cancers have also been reported to be highly expressed, but this high expression which does not lead to carcinogenesis raises questions about the role of oncogenes in carcinogenesis. The resistance of cancer cells to drugs, apoptosis, and immune cells does not rely solely on oncogene overexpression but also on the defect in cell organelle machinery (mitochondria, endoplasmic reticulum, and cytoskeleton). This review discusses factors contributing to cancer; we report the dysfunction of the cell organelles and their contribution to carcinogenesis, while oncogene overexpression promotes tumorigenesis, maintenance, and progression through cell adhesion. All these factors together represent a fundamental requirement for cancer and its development.

## Introduction

Glioma is the deadliest tumor of the central nervous system. The increasing number of patients and the complex risk factors have shown that this disease evolves and changes form (Zhang et al., 2016). Despite the advanced discovery on cancer initiation and the manifestations in the study of cancer stem cells, the mechanism of action, and the origin of glioma is still a holy grail of cancer research (Archer et al., 2011; Sarkar and Hochedlinger, 2013; Zhang et al., 2016, 2019). Several factors are implicated in glioma through the unusual dysfunction of the brain cell organelles, a physiological and genetic phenomenon that occurs in a single cell to induce the transformation, and uncontrollable multiplication of a single cancer cell into a complex cellular system (tumor) that escapes immune system control (Alcantara Llaguno and Parada, 2016). How a single cell can accumulate all of the required genetic mutation and epigenetic modification is still unclear. Despite the development of cellular investigation technologies like The Cancer Genome Atlas, next-sequence generation, cut and run, single-cell RNA sequencing demonstrated receptor tyrosine kinase (RTK)/RAS, P53 gene mutation, and their role in carcinogenesis and tumor development, still cancer remains a mystery of science, and not every mutation leads to cancer initiation (Imperial et al., 2019). The cell progression relies on gene expression, contact with other cells, and its microenvironment for its survival (Berg et al., 2002). A single isolated cell from its original tissue lacks all these advantages. Instead of dying, the single isolated cell activates a protection mechanism consisting of regulating its metabolism by regulating the activities of the mitochondria and by switching between aerobic and anaerobic glycolysis, the use of the exocytosis vesicles produced by the endoplasmic reticulum (ER) for its survival, and the regulation of cytoskeletal filament polymerization and depolymerization to infiltrate any tissue and change a cell type (Chen et al., 2012; Fife et al., 2014; Xiao et al., 2019). The single cell chooses its metabolite according to its environment and the availability of energy reserve (Janssen et al., 2019). The cells proliferate only when they have an acceptable quantity of accumulated energy and nutrients inside the cytoplasm (vacuole), so the overactivity of the mitochondria and the ER, combined with the upregulation of oncogenes, stimulates carcinogenesis, and tumorigenesis (Zhu and Thompson, 2019). These organelles and oncogenes synergistically activate specific signaling pathways that affect single-cell immortality. In this review, we will briefly discuss carcinogenesis and tumorigenesis based on the actions of the mitochondria, ER, and cytoskeletal stress response. We also delineate the functions of cancer-associated fibroblasts (CAF) as well as the contributions of the extracellular matrix (ECM) to carcinogenesis and tumorigenesis.

## Modification of the Mitochondrial Metabolic Activity in a Single Isolated Cell Correlates With Oncogene Upregulation and Energy Accumulation

Sex-determining region Y-box 1 (SOX1) is an oncogene known for its role in male sex and central nervous system differentiation (Archer et al., 2011). SOX1 plays several roles in brain cancer, including proliferation, invasion, migration, and survival (Garcia et al., 2017). The diverging opinion on SOX1 gene is mainly due to its two genes on chromosome 13 and Y. SOX1 can revive the central nervous system dead neurons and prevent them from dying by regulating their electrical impulse (Kanwore et al., 2021). We also attribute this function to the mitochondria, which played a key role in electron transport, and metabolism within neurons to promote the survival of an isolated cell. The role of SOX1 in mitochondrial activity, especially in metabolism, and has been elusive. Cell proliferation depends largely on cell metabolism and energy intake. The mitochondria have their own DNA (mtDNA) that encodes mitochondrial proteins, but the mitochondria also import nuclear proteins such as SOX1 (Stojanovski et al., 2003). Interestingly, the upregulation of SOX1 is involved in glioma malignancy and proliferation, but the mechanism involved in this process is not well defined. The mechanism that can explain the uncontrollable cell growth is energy Adenosine triphosphate (ATP) synthesis *via* regulation of the tricarboxylic acid (TCA) cycle and electron transport, cytoskeleton filament polymerization to enhance metabolic subtract trafficking, and high glucose intake and upregulation of the oncogene to stimulate cell cycle, and the survival of cancer cells. SOX1 promotes mitochondrial activity by combining itself with glucose 6-phosphate to enhance the TCA cycle and energy synthesis.

**FIGURE 1:**
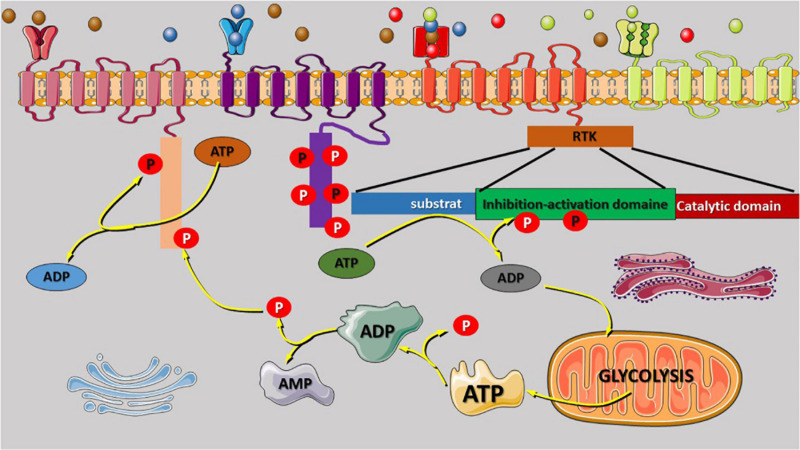
Receptor tyrosine kinase (RTK) regulatory domain auto-phosphorylation and phosphate origin. The binding of growth factors to the extracellular binding domain of the RTK receptor leads to activation and signal transmission to the intracellular domain that regulate the phosphorylation of several kinases. RTK regulatory domain auto-phosphorylation was reported to be involved in intracellular signal activation and transduction. Most of the phosphate atoms were derived from the dephosphorylation reaction of ATP into ADP and AMP. Glycolysis is the source of ATP synthesis that provides energy *via* the release of one atom of phosphate that can also participate to the phosphorylation reaction or enhance the activities of the Golgi, and the endoplasmic reticulum for protein (oncoproteins) exits *via* exocytosis to stimulate the other cell. AMP or ADP release during ATP dephosphorylation is recycled *via* the tricarboxylic acid cycle in the mitochondria. The variety of RTK family receptor and the modification of their extracellular domain can be activated by any binding molecules (unspecific) as shown in the figure (red receptor). The extracellular domain can be duplicated to increase the quantity of the receptor-specific binding molecules (green receptor).

In neoplastic cells, SOX1 binds to glucose 6-phosphate, which is transformed into pyruvate and acetyl-coA, and then transforms into energy (ATP) *via* the TCA cycle. Inside the mitochondria, SOX1 catalyzes major metabolism substrates of the TCA cycle, such as citrate, succinate, fumarate, malate, and oxaloacetate biodegradation to increase the TCA cycle turnover (Figure 1). Beyond this primary function, mitochondrial hyperactivity promotes mtDNA damage, resulting in abnormal morphology, decreased activity of cytochrome C oxidase, and high lactic acid production responsible for neuronal inflammation and cancer cell proliferation (Shiratori et al., 2019). Mutation or deletion of the mitochondrial genome can be transmitted from generation to generation (Howell et al., 2000). This mutation of the mitochondrial genome is responsible for isolated single-cell survival and autotrophy (Gilkerson et al., 2012). The increase in electron transport in the respiratory chains also contributes to energy synthesis because the dehydrogenase couple (NAD+/NADH or FAD+/FADH) responsible for electron transport participates in high energy release *via* the rupture of the energy bonds contained in their molecular structure (Sousa et al., 2018). SOX1 also enhances the mtDNA transcription inside the mitochondria by promoting the mitochondria transcription enzymes, such as mtDNA polymerase gamma (POLG). POLG is responsible for mtDNA transcription into RNA and the transformation of deoxyribose into mtRNA ribose (Nissanka et al., 2018). SOX1/POLG regulates the pentose phosphate pathway, which also contributes to the biogenesis of mtDNA and mtRNA, and maintains the single strand of mtDNA intact.

## The Endoplasmic Reticulum Structural Orientation in a Single Isolated Cell Enhances Its Survival and Promotes Cancer Initiation

Endoplasmic reticulum is an important organelle in protein biosynthesis, exocytosis vesicle (exosomes) formation, and protein exocytosis of proteins in the extracellular microenvironment (He et al., 2020). The traditional process of protein biosynthesis starts from the transcription of DNA into mRNA and then its translation into a protein that would then be evacuated out of the cell into a vesicle by exocytosis through the ER. In a single isolated cell, the ER changes its configuration to limit protein exocytosis to protect the cell from death (autotrophy). Cancer cells can switch their metabolism (anaerobic and aerobic) depending on the conditions of their environment. By changing the configuration of the ER, the cancer cell can switch its metabolism by increasing protein metabolism rather than glycolysis. The hyperdegradation of glucose in the mitochondria (high lactic acid synthesis) and the decarboxylation reactions lead to anhydrate and acidification reactions of the cytosol, thus modifying the configuration of the ER, and the ribosomes attached to its membrane. Cytosol acidification modifies the plasticity of cytoplasmic organelles and damages the ER DNA by activating ER-associated ribosomes (unification of subunits) to promote protein biosynthesis. The ribosome numbers on the ER are important for the isolated single-cell survival. The greater the population of ribosomes on the ER, the more ER stress in isolated single-cell survival. ER stress is a cause of several central nervous system-related diseases (Goto et al., 2018). It affects the neurons (microenvironment, metabolism, DNA/RNA, and plasticity, etc.) and alters the hormonal system that is the pillar of brain balance and functioning (Hosoi et al., 2018). Lebeaupin et al. (2020) and Cubillos-Ruiz et al. (2017) reported the role of ER stress in cancer initiation caused by oxidative stress, hypoxia, and lactic acidosis, significantly affecting cell dynamics, rescuing dead cells from apoptosis, and promoting their survival. The synergistic interaction of the ER and the mitochondrion in neuronal cells is believed to be the cause of its survival and the initiator of central nervous system cancers such as glioma. The ER can also contribute to mitochondrial metabolism by regulating the level of glucose inside the cell. During stress, the ER can convert lipids, proteins, and glucose 6-phosphatase dephosphorylation into glucose to be used by the mitochondria to produce energy.

## The Upregulation of Microtubule-Associated Protein Is Important for Molecular Signaling and for the Translocation of the Nucleus Protein to the Cytoplasm of Cancer Cells (*Vice Versa*)

The cytoskeleton is made up of microtubules, intermediate, and microfilaments. The interlinking of these protein filaments occupies a large part of the cell cytoplasm, extending to the cell nucleus. The cytoskeleton contributes to cell shape (morphology) and fortifies the cell with resistant features to extracellular forces (deformation and osmosis…), and it is involved in the connection between cells of the same tissue. The cytoskeleton is designed to facilitate cancer cell migration, import and export of molecules into cells, separation of chromosomes, and organization of space within cells. Cytoskeleton disruption was reported to be responsible for several diseases of the central nervous system, like gliomas, Parkinson’s disease, and Alzheimer’s disease, etc. The stability of the microtubule, the main filament of the cytoskeleton, is vital for the cell. In central nervous system cancer, the microtubule structure is compromised due to the upregulation of MAPs like DCX, Rho, and tau which regulate the microtubule organizing center responsible for microtubule filament polymerization by regulating the actin and tubulin concentrations inside the cell (Desai and Mitchison, 1997). The tubulin filament plays a key role in the polymerization and stabilization of the microtubule. The modification of tubulin configuration is a reason for cancer resistance to apoptosis because cancer cells can switch the polymerization of the microtubule by enhancing the expression of DCX. In association with GTP molecules, the phosphorylation of tubulin disrupts the stability of the microtubule, leading to cytoskeleton shrinking, depolymerization, and disassembly of microtubule proteins, with the production and release of high energy that cancer cells use for their survival. GTP to GDP hydrolysis mediated by DCX promotes cancer cell survival. Ayanlaja et al. (2020) explained the interactions between DCX and GTP in regulating DCX translocation in favor of glioma progression. This translocation of DCX from the cytoplasm to the nucleus *via* RanGTP can be reversible because RanGTP is a bidirectional protein transporter. The movement of DCX within cancer neurons is linked to the polymerization and depolymerization of the cytoskeleton, and enhances the survival of cancer cells. The translocation of DCX to the nucleus regulates cytoskeleton shrinkage, while DCX in the cell cytoplasm promotes the polymerization of the cytoskeleton due to high GTP, and a stabilizer of tubulin proteins. This mechanism explains why the findings reported by Ayanlaja et al. (2020) and Santra et al. (2009, 2010) are all correct because this translocation of DCX also relies on its expression. A high expression of DCX leads to its translocation to the nucleus, while DCX downregulation, and normal expression do not stimulate its translocation mechanism. The high level of GTP supports the polymerization and stability of the cytoskeleton. The reversible mechanism of DCX translocation reported by Ayanlaja et al. (2020) and Santra et al. (2010) showed that the goal of DCX in regulating the cytoskeleton as a therapeutic strategy will not be effective. A high expression of DCX promotes microtubule shrinkage and energy production *via* GTP hydrolysis to GDP. DCX downregulation or normal expression promotes cytoskeleton polymerization and stabilization using GTP. DCX promotes brain neuron survival but is not indispensable. Moreover, in cancer, DCX not only regulates cancer progression. The study by Ayanlaja et al. (2020) and Santra et al. (2010) showed both sides of DCX in cancer progression. DCX downregulation in cancer stem cells showed that DCX is important for neuronal cancer stem cell differentiation *via* epithelial–mesenchymal transition gene regulation that will contribute to tumor bulk. The best therapeutic strategy is targeting of stem cell marker genes (SOX2, SOX1, and vimentin, etc.) to reduce the population of cancer stem cells.

## Gene Alteration: Metabolism Stimulates Auto-Phosphorylation of Receptor Tyrosine Kinase to Amplify Cell Signaling

It was reported that RTK consists of four domains that connect them to their ligands (extracellular) and substrate (intracellular). The first domain is the extracellular ligand-binding domain, which allows a specific connection between the receptor to the ligands (key in lock). The conserved catalytic protein tyrosine kinase domain specializes in intracellular signal transduction, the regulatory domain is known for phosphorylation reactions, and the transmembrane domain connects the ECM to the intracellular domain (Li and Hristova, 2006). The regulatory domain plays a critical role in the transduction of signals regulated by the intracellular ligand-binding domain. The aberrant alteration of the regulatory domain can enhance the phosphorylation reaction without binding the ligand on the extracellular ligand-binding domain and promote molecular inter-phosphorylation by stimulating the cytoplasmic catalytic domain to promote signal transduction. The auto-phosphorylation induced by the regulatory domain can affect the phosphorylation of the cytoplasmic substrate, modifying cell biological and physiological functions such as metabolism, survival, and cellular proliferation (Figure 1; Spangle and Roberts, 2017). It is well known that PI3K/AKT/mTOR regulates the aerobic and anaerobic glycolysis in the mitochondria (Makinoshima et al., 2015; Hu et al., 2016; Xie et al., 2019). The effect of PI3K/AKT/mTOR on the epigenetic modifications, such as histone H3K4 and H3K9 acetylation or hypermethylation, and affects gene expression modification at the promoter region. The hypermethylation of the oncogene promoter region increases their expression level, while the methylation of the silencer region does not have a significant effect on oncogenes. The next-generation sequence and single-cell sequencing technologies have improved our understanding of gene mutations and epigenetic modifications involved in cancer initiation and development through mutational activation, oncogene amplifications, and inactivation of tumor suppressor genes. Song et al. (2011) reported that the acetylation of RTK gene at k684, k836, and k843 by creb-binding protein, an acetyltransferase enzyme, modulates its activity as well as other receptors [epidermal growth factor receptor (EGFR) and VGFR], leading to receptor overactivation, multiple signal transduction, and oncogene overexpression.

## RTK Signaling Is Critical for Neuron–Axon Regeneration in Central Nervous System Cancer and Tumor Progression

Rreceptor tyrosine kinase was reported to affect several physiological functions, starting with cell division, differentiation, survival, adherence, migration, and apoptosis (Schlessinger, 2000; Beyer and MacBeath, 2012). The RTK family encoded up to 20 subfamilies, including growth factor receptors such as EGFR, nerve growth factor receptor (NGFR), and fibroblast growth factor receptor (van der Geer et al., 1994; Robinson et al., 2000). RTKs regulated the central nervous system axon regeneration to promote neuron connection *via* axons to reduce neuron degeneration (Vigneswara et al., 2012). The axon regeneration depends on the interaction of RTKs with EGFR overlapping signal transduction. Multiple structural RTK domains favor the binding of extracellular RTK ligands to their family receptor (EGFR and NGFR) to regulate intracellular signal transduction by the activation of the RTK phosphorylation regulatory domain, leading to the phosphorylation of its substrate. The phosphorylation of RTK substrates leads to the activation and regulation of various signaling pathways such as PI3K and RAS/MAP. However, the activation and phosphorylation of RTK can induce the physiological function of the cell. A positive signal activation of RTK promotes the cell cycle and the differentiation of the central nervous system, while a negative signal reduces the cell cycle and differentiation (Dikic and Giordano, 2003). Boyd and Gordon (2001) and Baker et al. (2007) reported that the binding of brain-derived neurotrophic factor (BDNF) to tyrosine receptor kinase B (TrkB) promotes regeneration of the axon of the dopaminergic neuron during brain injury, synaptogenesis, and central nervous system growth (Boyd and Gordon, 2001; Baker et al., 2007). TrkB and BDNF were also reported to regulate AKT, MAPK, and STAT3 signaling pathways to enhance neuronal stem cell multiplication and differentiation into a variety of central nervous system neurons (Islam et al., 2009). Based on all this information, RTKs are strategic kinases that regulate the central nervous system cell functions, survival, and neuronal stem cell differentiation by switching the signaling pathways and substrates.

## Extracellular Serpin 1 Regulates TP53 Inactivation, Retinoblastoma Protein Upregulation to Stimulate Senescent Neuron Proliferation and Brain Cancer

The cell environment is important for its survival. PIA-1 is an extracellular protein that regulates cell functions, especially cellular senescence. Increased extracellular PAI-1 expression induces single isolated cell senescence and apoptosis through the upregulation of P53 and its inactivation, leading to lysosome and phagosome activation, and to cell autophagy to promote isolated single-cell apoptosis (Xie and Klionsky, 2007; Csizmadia and Juhasz, 2020). Autophagy is important for non-starved cell homeostasis (Djajadikerta et al., 2020). The defect of cell autophagy leads to metabolism changes in various central nervous system diseases such as cancer (Levy et al., 2017; Djajadikerta et al., 2020). A defect autophagy of brain neurons leads to the survival of senescent cells that can promote brain cancer. TP53 or P53 is a tumor suppressor gene that regulates genetic material (DNA and RNA) repair. It was reported that most of the human tumors contain P53 gene mutation induced by environmental factors (Greenblatt et al., 1994; Hussain and Harris, 1998). P53 function inactivation due to genomic alteration or its promoter hypermethylation is frequently observed in carcinogenesis (Newcomb et al., 1993; Nozaki et al., 1999). CpG dinucleotides are commonly found near gene promoters and are subject to genetic material methylation (Luczak and Jagodzinski, 2006). Aberrant CpG hypermethylation was reported to be involved in carcinogenesis. Hypermethylated CpG binding to the tumor suppressor promoter region was reported to promote gene transcription suppression (Esteller, 2002). Pfeifer (2000) reported the role of methylated CpG in P53 gene mutation and inactivation associated with carcinogenesis. The deamination of 5-methylcytosine is the main cause of P53 gene mutation in human internal cancers due to the replacement of cytosine (C) with thymine (T) at CpG sites, contributing to increased mutation rate, and chromosome microsatellite instability in tumors (Gonzalgo and Jones, 1997; Riccio et al., 1999). Inactivation of the P53 gene without alteration suppresses cell cycle arrest, senescence, and cell apoptosis (Aylon and Oren, 2011). Under normal conditions, P53 regulates cell cycle arrest *via* the retinoblastoma gene known to promote cyclin family protein binding and phosphorylation during the cell cycle. Inactivation of P53 results in RB phosphorylation and its separation with E2F unit, encouraging uncontrollable cell cycle progression, and DNA hypertranscription (Lees et al., 1993; Giacinti and Giordano, 2006). TP53 isoforms also contribute to tumorigenesis. The overexpression of TP53α, TP53β, and TP53δ has been reported to reduce cancer cell sensitivity and resistance to chemotherapeutic drugs. TP53α and TP53β ratios play a critical role in glioma cell proliferation and migration (Bisio et al., 2013; Su et al., 2015). When the ratio TP53α/TP53β is lower than one, it promotes TP53 polymorphism and IRES mutation/splice site, leading to inflammation, glioma cell proliferation, migration, and angiogenesis to stimulate tumorigenesis and cancer progression. If the ratio TP53α/TP53β is higher than one, it promotes alternative splicing, chromosomal degradation, and epigenetic alteration of oncogenes, leading to brain cell senescence, enhanced neuronal physio-pathology, and gene transcription that can stimulate carcinogenesis (Andreotti et al., 2011; Bisio et al., 2013; Lion et al., 2015; Su et al., 2015). P53 is a pivotal gene that is connected to several signaling pathways that regulate the cell cycle.

## The Niche: CAFs Initiation and Glioma Stem Cell Proliferation, Invasion, and Metastases

The niche is a specific site of a tissue containing nutrients in which the cells thrive. The composition of the niche is essential for cell development and differentiation. They are composed of fibroblasts, immune, endothelial, and perivascular cells, ECM, cytokine network, and growth factors (Birbrair and Frenette, 2016; Yu and Scadden, 2016). The niche composition contributes to tumor cell heterogeneity, tumor progression, genetic diversity, and epigenetic modifications (Ho and Shim, 2017; Aderetti et al., 2018). Thus, any change in the niche composition affects the cell cycle and ability to differentiate. Specifically, changes in the composition of the fibroblasts are common in the stem cell niche and can synthesize the ECM (glycosaminoglycans and reticular), collagen (collagen fibers), and connective tissue in animals (elastic fibers). Fibroblasts are the primary immune response cell triggered during microorganism invasion and inflammation (Dave et al., 2004; Silzle et al., 2004; Bonnans et al., 2014). It is therefore crucial to understand the mechanism of action and the sequence of events in response to immunoreactivity.

Glial cell line-derived neurotrophic factor (GDNF) is highly expressed in all types of gliomas, regardless of grade or form (Ayanlaja et al., 2018), and this expression pattern contributes to the recruitment and activation of caner-associated fibroblasts, leading to an inflammatory response (Tape et al., 2016). The activation of the immune response and the corresponding inflammatory reactions promote the synthesis of interleukins (IL-6/8) and CD113 by T-cells for repairs (Tanaka et al., 2014). The overexpression of IL-6 and IL-8 can further activate Pi3k/Akt and hypoxia-inducible factor-alpha to influence cell proliferation and brain neuron inflammation, and modifying the neuronal cell microenvironment (Santos and Andrade, 2017). During hypoxia, there is a surge in NFkβ expression, which promotes neuronal senescence and apoptosis *via* autophagy. However, in cancer stem cells, the same process promotes survival through oncogene-induced CAF activation to prevent mesenchymal cell death and promote the survival of senescent cells in the brain (Guryanova et al., 2011; Shan et al., 2015). These oncogenes, such as GDNF, STAT3, and SOX1, induce CAF activation and promote the overexpression of growth factors such as CXCL12 and Wnt/Notch for differentiating epithelial cells into tumor-initiating stem cells (Hirano et al., 2000; Shan et al., 2015). The proliferation of CAF cells affects neural stem cell morphology, modifies the microenvironment, and impacts on mesenchymal stem cells and their implications for glioma stem cell proliferation and metastasis (Shan et al., 2015; Zhang et al., 2017). Furthermore, improved glioma stem cell proliferation promotes immunosuppression in the niche by secreting more cytokines and chemokines, subsequently inhibiting immune cell response (Hirano et al., 2000; Tanaka et al., 2014). Immune cells are the regulator of cell growth competition in the niche. Their inhibition led to uncontrollable cell proliferation, especially CAFs, which represent 90% of tumor bulk (Feig et al., 2012).

## Modifications in Cellular Matrix Interactions and Cell–Cell Contact Promote the Initiation of Glioma

Cell-to-cell interaction is indispensable for its survival. A single isolated cell eventually loses its functional capacity and dies of apoptosis because it no longer receives stimulation (hormones and proteins) from its source tissue. This reaction increases the risk of cancer by inducing metalloproteinase-3 (MMP3) expression, which leads to genomic instability, mitochondrial shape modification, and metabolism degradation, leading to a high caspase-3 expression induced by cytochrome C. The increase in extracellular MMP3 particularly promotes Bcl2 binding to the mitochondria and the upregulation of mitochondrial outer membrane permeability, and disrupting the mitochondrial membrane (Kale et al., 2018). The mitochondrial membrane disruption normally leads to its destruction by lysosomes, but the destruction of damaged mitochondria for recycling stopped the massive inwardness of SOX1 in the mitochondria to rescue damaged mitochondria from lysosomes. The upregulation of SOX1 in the damaged single isolated and suspended cells promotes the cell type transition and transforms the cell into a cancer stem cell, the main cause of tumor formation, and cell proliferation. A small modification in the niche significantly affects cell–cell contact and tumor initiation *via* the secretion of neuroprotective proteins like GDNF or hormones (dopamine) and cancer stem cell markers (Anderson et al., 2007). GDNF overexpression can also activate CAFs and support cells like astrocytes to rescue damaged (DNA, ER, and mitochondria) or senescent neurons by cell–cell contact using cytoskeleton and cytoplasmic extensions. The CAFs may bind to senescent cells and initiate their transformation into tumor cells (Trylcova et al., 2015; Zhang et al., 2017).

**FIGURE 2:**
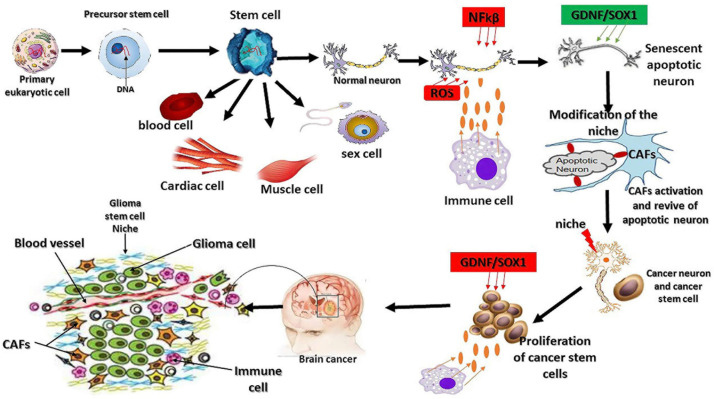
Mutation origin and the factors regulating glioma initiation. As we know today, eukaryotic cells originate from the symbiotic association between a host cell and microorganism (archeae bacteria). This physical association leads to genetic mutation, synthesis of novel proteins, and cell structure modification. Cancer or glioma cells also behave like primary eukaryotic cells by modifying their structure, gene mutations, and aberrant synthesis of a novel oncogene. The extrinsic factors involved in the oxidative stress reactions promote the overexpression of NFkβ and interleukin 1α to initiate cell death and senescence. The response to oxidative stress reaction triggers the synthesis of certain oncogenes, such as GDNF and SOX1, and the subsequent activation of fibroblast cells to revive the senescent cells, leading to the dysfunction of the ability of the revived neurons to maintain their living status *via* the overexpression of GDNF and SOX1 because of their neuroprotective effects and their protection by immune cells (Ayanlaja et al., 2018). Stem cells are the origin of living tissues *via* differentiation into many types of cells; however, they are not necessarily the origin of glioma mutations as this phenomenon is inherited from the primary eukaryotic cell. The other factors influencing stem cell differentiation are the niche and other extrinsic factors.

The desmosome is a region where the plasma membrane adheres to another cell plasma membrane (Delva et al., 2009). The overexpression of SOX1 aids this interaction (cell–cell contact) *via* the regulation of β-catenin expression to promote CTNNA2 and CTNNA3 gene overexpression, which are involved in cell–cell adhesion (Dong and Ubogu, 2018; Schaffer et al., 2018). The overexpression of CTNNA2/3 negatively regulates ARP2/3 activity that promotes actin polymerization to control the morphological changes of isolated cells and improve cell–cell contact (Schaffer et al., 2018). SOX1 interacts with CTNNA2/3 by targeting the gene on chromosome 2 at P12 locus to stimulate the upregulation of collagen and keratin and subsequently improves cell adhesion through the desmosome, whereas the polymerization of the cytoskeleton will pressurize and push the plasma membrane closer toward glioma cells or normal cell during the invasion, and formation of the glioma stem cell colony as well as tumor growth *via* activated CAF cells. CAFs facilitate cancer cell migration by regulating the organization of fibronectin and collagen fibers (Figure 2; Erdogan et al., 2017).

## Conclusion

Cancer initiation relies on the internal reorganization of the isolated cell organelles regulated by the cytoskeleton (Kim and Cheong, 2020; Ong et al., 2020). The cell organelles play an important role in cellular functions and survival. The mitochondria or ER damages contribute to the development of diseases by modifying the metabolic substrates to ensure the survival of an isolated cell and adaptation to its new environment. Cell metabolism regulates several mechanisms and machinery to maintain the energy level in all its forms to protect the cell against apoptosis (Zhu and Thompson, 2019). The failure of this machinery activates either cell death or senescence mediated by autophagy, which can rescue the cell from death through the recycling of unwanted organelles and activation of oncogenes (Glick et al., 2010). Oncogenes such as SOX1, GDNF, and DCX strongly maintain this machinery to ensure an the survival and proliferation of the isolated cell. Oncogene alteration also relies on the extracellular environment like the cell niche; the ECM can modify cell function by regulating the gene expression. Oncogene mutation or epigenetic modification ensures the maintenance and progression of cancer, but the initiation strongly relies on the organelles and their ability to maintain cell integrity, and energy at an acceptable level to prevent cell death or senescence. Brain cancer should be a defect in organelles that control cell metabolism and energy synthesis necessary for cell cycle.

## Author Contributions

KK was the mastermind behind the ideas, write-up, and image production. X-XG helped in data gathering. AA was responsible for reorganization and editing. PK was responsible for further data gathering. YX and DG contributed to read and approved the manuscript for publication. All authors contributed to the article and approved the submitted version.

## Conflict of Interest

The authors declare that the research was conducted in the absence of any commercial or financial relationships that could be construed as a potential conflict of interest.

## Publisher’s Note

All claims expressed in this article are solely those of the authors and do not necessarily represent those of their affiliated organizations, or those of the publisher, the editors and the reviewers. Any product that may be evaluated in this article, or claim that may be made by its manufacturer, is not guaranteed or endorsed by the publisher.

## Abbreviations

CNS: central nervous system
GSC: glioma stem cell
NSC: neuronal stem cell
DNA: deoxyribonucleic acid
RNA: ribonucleic acid
GDNF: glial cell line-derived neurotrophic factor
SOX1: sex-determining region Y box1
ROS: oxygen stress response
STAT3: signal transducerand activator of transcription 3
DNMT: DNA methyltransferase
MMP3: metalloproteinase-3
ECM: extracellular matrix
CAF: cancer-associated fibroblast
TISC: tumor-initiating stem cells
CTNNA2: catenin alpha 2
IL-6: interleukin
GFL: glial cell line-derived neurotrophic family ligands
redox: oxidation–reduction.
